# Horizontal Transfer of Symbiosis Genes within and Between Rhizobial Genera: Occurrence and Importance

**DOI:** 10.3390/genes9070321

**Published:** 2018-06-27

**Authors:** Mitchell Andrews, Sofie De Meyer, Euan K. James, Tomasz Stępkowski, Simon Hodge, Marcelo F. Simon, J. Peter W. Young

**Affiliations:** 1 Faculty of Agriculture and Life Sciences, Lincoln University, P.O. Box 84, Lincoln 7647, New Zealand; simon.hodge@lincoln.ac.nz; 2 Centre for Rhizobium Studies, Murdoch University, Murdoch 6150, Australia; s.demeyer@murdoch.edu.au; 3 Laboratory of Microbiology, Department of Biochemistry and Microbiology, Ghent University, 9000 Ghent, Belgium; 4 James Hutton Institute, Invergowrie, Dundee DD2 5DA, UK; euankevin.james@hutton.ac.uk; 5 Autonomous Department of Microbial Biology, Faculty of Agriculture and Biology, Warsaw University of Life Sciences (SGGW), 02-776 Warsaw, Poland; sttommic@yahoo.co.uk; 6 Embrapa Genetic Resources and Biotechnology, Brasilia DF 70770-917, Brazil; marcelo.simon@embrapa.br; 7 Department of Biology, University of York, York YO10 5DD, UK; peter.young@york.ac.uk

**Keywords:** Fabaceae, lateral gene transfer, legumes, N_2_ fixation, nodulation, *nod* genes

## Abstract

Rhizobial symbiosis genes are often carried on symbiotic islands or plasmids that can be transferred (horizontal transfer) between different bacterial species. Symbiosis genes involved in horizontal transfer have different phylogenies with respect to the core genome of their ‘host’. Here, the literature on legume–rhizobium symbioses in field soils was reviewed, and cases of phylogenetic incongruence between rhizobium core and symbiosis genes were collated. The occurrence and importance of horizontal transfer of rhizobial symbiosis genes within and between bacterial genera were assessed. Horizontal transfer of symbiosis genes between rhizobial strains is of common occurrence, is widespread geographically, is not restricted to specific rhizobial genera, and occurs within and between rhizobial genera. The transfer of symbiosis genes to bacteria adapted to local soil conditions can allow these bacteria to become rhizobial symbionts of previously incompatible legumes growing in these soils. This, in turn, will have consequences for the growth, life history, and biogeography of the legume species involved, which provides a critical ecological link connecting the horizontal transfer of symbiosis genes between rhizobial bacteria in the soil to the above-ground floral biodiversity and vegetation community structure.

## 1. Introduction

Approximately 70% of the ca. 19,300 species in the Fabaceae (Leguminosae, the legume family) can fix atmospheric nitrogen (N_2_) via symbiotic bacteria (general term ‘rhizobia’) in root nodules [1,2]. Rhizobia reduce atmospheric N_2_ to ammonia (NH_3_) through the enzyme nitrogenase, and this NH_3_, as ammonium (NH_4_^+^), is transported to plant cells where it is assimilated into amino acids via the glutamine synthetase/glutamate synthase (GS/GOGAT) pathway [3,4]. The ability to fix N_2_ can give legumes an advantage under low soil nitrogen (N) conditions if other factors are favourable for growth [5,6]. Also, legume N_2_ fixation can be a major input of N into a wide range of natural and agricultural ecosystems [7,8,9,10].

The most recent classification of the legumes identifies six legume sub-families, namely, the Caesalpinioideae, Cercidoideae, Detarioideae, Dialioideae, Duparquetioideae, and Papilionoideae, but only species within the Caesalpinioideae and Papilionoideae nodulate [1]. In this classification, the sub-family Caesalpinioideae includes all members of the former sub-family Mimosoideae (now referred to as the Mimosoid clade), which contains most of the nodulating legumes within the Caesalpinioideae. For most legumes, the nodulation process is initiated by the legume production of a mix of compounds, mainly flavonoids, which activate nodulation protein D (NodD) in rhizobia by stimulating the binding of NodD to *nod* gene promoters [11,12]. Different legumes produce different types and mixes of compounds, and this can be a point of legume rhizobium symbiosis specificity [13]. The NodD protein triggers the transcription of a range of genes within the rhizobium, including those required to produce Nod factors, the signal molecules from the rhizobium which induce nodule morphogenesis in the legume [14]. These genes include *nodABC* which encode the enzymes required for the synthesis of the core Nod factor structure of an *N*-acetyl glucosamine oligosaccharide backbone with a fatty acyl chain at the non-reducing end [12]. Nod factors differ in their length of the *N*-acetylglucosamine oligosaccharide backbone and length and saturation of the fatty acid chain. Other *nod* genes encode species-specific modifications to the Nod factor structure [12], and, related to this, specific *nod* genes have been shown to be major determinants of legume host specificity [15,16]. Rhizobia enter the roots of most legume species so far studied via root hair infection [2]. Host cell wall material grows around the developing ‘infection’, forming an infection thread which grows through the root cortex, branching repeatedly. Rhizobia are released from the tips of these infection threads into membrane-bound structures within the legume cells, called symbiosomes, where they differentiate into their N_2_-fixing form known as bacteroids in root nodules. Bacteroids differ in their level of differentiation and viability, and nodules can be indeterminate or determinate in growth, depending on the legume host [2,17,18]. Indeterminate nodules maintain meristematic activity, while determinate nodules have a transient meristem. All genera examined in the Caesalpinioideae and most tribes within the Papilionoideae had indeterminate nodules, but the Dalbergieae, Desmodieae, Phaseoleae, Psoraleeae, and some members of the Loteae had determinate nodules [2].

The *nod* genes and the *nif* genes, which encode the subunits of nitrogenase, are often carried on symbiotic islands or plasmids that can be transferred (horizontal—lateral—transfer) between different bacterial species within and across genera [19,20,21,22]. The symbiosis genes involved in horizontal transfer have phylogenies different from those of the core genome of their ‘host’ [17,22]. Here, the literature on legume–rhizobium symbioses in field soils was reviewed, and cases demonstrating incongruence between rhizobium core and symbiosis genes were collated. The occurrence and importance of horizontal transfer of rhizobial symbiosis genes within and between bacterial genera were assessed.

## 2. Framework and Assumptions of the Study

Rhizobia were aligned with their legume symbionts according to legume sub-family, tribe, genus, and nodule type. Where examined, bacteroids of genera in the Inverted Repeat-Lacking Clade (IRLC, Papilionoideae) were terminally differentiated and could not return to their bacterial form [2,23]. Also, several species in the IRLC were shown to have a high degree of rhizobial specificity related to specific symbiosis genes that have been shown to be transferred between rhizobial species [17], and this clade was considered separately.

The rhizobia genera used in the search were those validated in the International Journal of Systematic and Evolutionary Microbiology. These are the alpha proteobacterial genera in the families Rhizobiaceae (*Rhizobium*, *Ensifer*/*Sinorhizobium*, *Allorhizobium*, *Pararhizobium*, *Neorhizobium*), Bradyrhizobiaceae (*Bradyrhizobium)*, Phyllobacteriaceae (*Mesorhizobium*, *Phyllobacterium*), Methylobacteriaceae (*Methylobacterium*, *Microvirga*), Brucellaceae (*Ochrobactrum*), Xanthobacteraceae *(Azorhizobium)*, Hyphomicrobiaceae *(Devosia*), and the betaproteobacterial genera in the family Burkholderiaceae (*Paraburkholderia*/*Burkholderia*, and *Cupriavidus*) [17].

**Figure 1:**
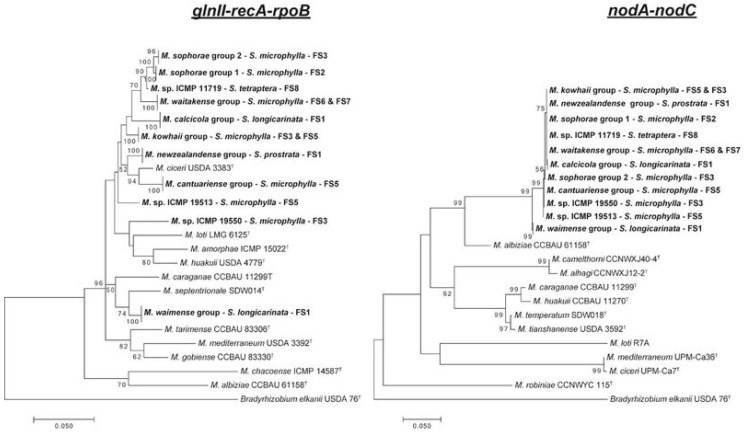
Comparative maximum likelihood phylogenetic analysis using housekeeping and symbiotic gene clusters from *Mesorhizobium* strains isolated from New Zealand endemic *Sophora* spp. Sequence alignment, alignment editing, and phylogenetic analysis were performed using MEGA7 [24]. The phylogenetic trees were built using the GTR model with G + I substitutions for the housekeeping genes (1083 bp) and Tamura 3-parameter model with G + I substitutions for the symbiosis genes (869 bp). The possibility of concatenation was investigated using the partition-homogeneity test with PAUP [25,26]. Each concatenation was investigated for 1000 replicates. All housekeeping genes were congruent with each other (*p* = 0.015), and both symbiosis genes were congruent with each other (*p* = 0.02). The housekeeping genes were shown not to be congruent with the symbiosis genes (*p* = 0.001). Bootstrap values after 500 replicates are expressed as percentages; values less than 50% are not shown. The scale bar indicates the fraction of substitutions per site. M: *Mesorhizobium*, S: *Sophora*, FS: Field Site.

A comprehensive collation of published cases of phylogenetic incongruence between rhizobium core and symbiosis genes until 30 September 2017 was carried out. Articles were collected by searching the Institute for Scientific Information (ISI) Web of Science, using each legume genus known to nodulate partnered with each of the rhizobia genera, and each of the rhizobia genera partnered with ‘horizontal gene transfer’ and ‘lateral gene transfer’ as key words. Further searches were carried out on the literature quoted in the selected papers and on those papers listed as quoting the selected papers in ISI Web of Science. Only data for plants sampled under field conditions, for plants grown in soils taken from the field, or for plants supplied field soil extracts, were used. Bacteria isolated from legume nodules were accepted as rhizobia on the basis of the criteria described previously [17]. All cases of ‘authenticated’ rhizobia for which core and symbiosis gene sequences were presented were studied further, and the cases for which the authors considered there was incongruence between core and symbiosis gene sequences are discussed. The core and symbiosis genes that showed incongruence are given in Tables. Representative data are presented for *Glycine max* and *Phaseolus vulgaris* because of the large number of publications reporting incongruence between core and symbiosis gene sequences for these two species. In some studies, incongruence was tested statistically but, in most cases, this was determined from a visual assessment of phylogenetic trees of the two sets of genes. For example, *Mesorhizobium* strains isolated from New Zealand endemic *Sophora* spp. had diverse concatenated (gene sequences aligned head to tail) *glnII-recA-rpoB* gene sequences but similar concatenated *nodA–nodC* gene sequences (Figure 1), and, here, the housekeeping and symbiosis genes were considered incongruent [17]. Subsequent statistical analysis of these sequences indicated that the housekeeping and symbiosis genes were incongruent.

## 3. Lateral Transfer of Symbiosis Genes

### 3.1. Rhizobia Associated with the Caesalpiniodeae

There are reports of phylogenetic incongruence between core and symbiosis genes for *Ensifer*, *Cupriavidus*, *Burkholderia*, *Rhizobium*, and *Devosia* associated with legumes in the Mimosoid clade of the sub-family Caesalpinioideae (Table 1).

Five separate studies indicated that horizontal transfer of symbiosis genes had occurred between *Ensifer* spp. associated with species in the Caesalpinioideae [27,28,34,35,36]. For *Ensifer* associated with the Mexican native *Leucaena leucocephala* sampled in Panxi, China, gene sequences indicated that symbiotic genes of strains associated with introduced plants were transferred into indigenous strains in the soil [28]. *Ensifer* isolates from *Vachellia macracantha* sampled within the plant’s native range in Peru showed *nifH* and *nodC* sequences closely related to other American rhizobial strains, which adds support to the use of symbiotic genes as valuable indicators of geographical origin [35]. Gene sequences indicated diverse origins for the housekeeping genes *nifH* and *nodA* for seven *Ensifer* isolates representative of 73 isolates from *Vachellia jacquemontii* sampled within its native range in the Thar Desert of India [34]. The authors suggested that the stressful desert conditions, and stressful conditions in general, may favour frequent horizontal gene transfer. Alternatively, rather than promote its occurrence, stressful environments may represent a situation where the positive consequences of horizontal gene transfer in terms of natural selection are more significant, and thus horizontal gene transfer becomes more apparent within the rhizobial population. 

Ten *Cupriavidus* rhizobia strains isolated from five *Mimosa* spp. in southern Uruguay showed symbiosis and housekeeping gene sequence phylogenies that were not congruent [29]. The strains separated into two groups of five strains on their 16S rRNA sequences, and one strain selected from each of these groups differed substantially in its *recA* and *gyrB* sequences. However, both the *nodA* and *nifH* sequences for the ten strains grouped together in a cluster. Also, *Rhizobium altiplani* Br 10423^T^ isolated from *Mimosa pudica* in Distrito Federal in central Brazil had *nifH* and *nodC* sequences closely related (identical for *nodC*) to those of *Rhizobium mesoamericanum* CCGE 501^T^ [32]. 

*Mimosa* is a genus of ca. 550 species native to the Americas, South Asia, and Africa including Madagascar [1,2]. The evidence indicates that *Cupriavidus* is the main rhizobial symbiont of endemic *Mimosa* in southern Uruguay, but *Burkholderia* and *Rhizobium*/*Ensifer* are the main symbionts of *Mimosa* in central and southern Brazil and central Mexico, respectively [29,37,38,39]. In contrast with findings for *Mimosa Cupriavidus* symbionts in Uruguay, the symbiosis gene sequences for *Burkholderia* in Brazil and *Rhizobium*/*Ensifer* in Mexico were largely congruent with their respective 16S rRNA and housekeeping gene sequences [37,38]. This indicates that these symbiosis genes diverged over a long period within *Burkholderia* and *Rhizobium*/*Ensifer* without substantial horizontal transfer between species. Similarly, it was concluded that *nodC* and *nifH* sequences for *Burkholderia* isolated from the *Piptadenia* group (*Piptadenia*, *Parapiptadenia*, *Pseudopiptadenia*, *Pityrocarpa*, *Anadenanthera*, and *Microlobius*) (Mimoseae) have evolved mainly through vertical transfer, with rare occurrence of horizontal transfer [40]. Outside South America, *Burkholderia* strains isolated from *Mimosa diplotricha* and *M. pudica* in Yunan province in subtropical China showed diverse 16S rRNA sequences but grouped together, along with *Burkholderia phymatum* STM815^T^, on *nodA* sequences [31]. Also, *Burkholderia caribensis* TJ182 isolated from the invasive *M. diplotricha* in Taiwan and characterized on 16S rRNA sequence, grouped with *Cupriavidus* strains on *nodA* sequence, indicating that the *nodA* gene had been transferred from *Cupriavidus* to *Burkholderia* [30]. However, this transfer was not confirmed [41]. In both studies, it was concluded that it was likely that the *Burkholderia* ‘travelled’ with their invasive hosts from South America to South East Asia [30,31].

A more extreme case of incongruence between core and symbiosis genes was reported from India for *Devosia natans* isolated from the aquatic ‘water mimosa’ *Neptunia natans*. Here, the 16S rDNA sequences indicated that two strains isolated from *N. natans* were *Devosia*, but the *nifH* and *nodD* sequences were most closely related to those of *Rhizobium tropici* CIAT899^T^ [33]. This finding indicates that the horizontal transfer of symbiosis genes has occurred across genera from *Rhizobium* to *Devosia* at some stage.

### 3.2. Rhizobia Associated with the Papilionoideae

#### 3.2.1. The Inverted Repeat-Lacking Clade (IRLC)

*Ensifer*, *Mesorhizobium*, and *Rhizobium* are the main rhizobial symbionts of legumes in the IRLC, and there are several examples of phylogenetic incongruence between core and symbiosis genes for *Mesorhizobium* and *Rhizobium* associated with IRLC species (Table 2).

Incongruence between housekeeping and symbiosis gene sequences indicates that within-genus horizontal transfer of symbiosis genes has occurred between *Mesorhizobium* strains associated with *Cicer arietinum* [42,43,44,45,46,47], *Cicer canariense* [48], *Astragalus glycyphyllos* [56], *Glycyrrhiza uralensis* [57], *Sphaerophysa salsula* [58], *Alhagi sparsifolia* [59], and five *Caragana* spp. [60], and between *Rhizobium* strains associated with Fabeae spp. [49,50,51,52,53,54], and *Trifolium* spp. [61,62] (Table 2). Considering the crop plants, evidence is strong that indigenous *Mesorhizobium muleiense* in Northwest China obtained its *C. arietinum* (chickpea) specific symbiotic genes from *Mesorhizobium ciceri* or *Mesorhizobium mediterraneum* associated with imported *C. arietinum* used as a crop [46]. Similarly, 83 isolates from *Trifolium repens* (white clover) grown in alkaline soils in subtropical China, identified as *Rhizobium anhuiense*, *Rhizobium leguminosarum*, and a novel *Rhizobium* genospecies on 16S rRNA and housekeeping gene sequences, had *nifH* and *nodC* sequences similar to *R*. *leguminosarum* sv. *trifolii* introduced with the crop. This indicates that the symbiosis genes had been transferred from the *R*. *leguminosarum* sv. *trifolii* strain to the native soil bacteria [62]. 

In Xinjiang, China, isolates of *Rhizobium multihospitium* were obtained from a number of plant species from different tribes: *Lathyrus odorata*, *Vicia hirsuta* (Fabeae), *Astragalus aksuensis*, *Astragalus* sp., *Oxytropis glabra*, *Oxytropis meinshausenii* (Galegeae), *Alhagi* sp., *Caragana jubata*, *Halimodendron halodendron* (Hedysareae) (Table 2); *Robinia pseudoacacia* (Robineae), *Sophora alopecurioides* (Sophoreae) (Table 3); *Lotus frondosus* and *Lotus tenuis* (Loteae) (Table 4). The *nifH* and *nodD* sequences of these isolates were 100% similar to those of *Rhizobium lusitanum* P1–7^T^ and *D. neptuniae* J1^T^ [55], and it was suggested that *nifH* and *nodD* genes of the three rhizobial species may have the same origin. Also, isolates from *S. salsula* identified as *Rhizobium* genotypes on 16S rRNA gene sequences showed similar *nifH* sequences to those of the *Mesorhizobium* isolates, while a *Bradyrhizobium* isolate (16S rRNA) from *Caragana intermedia* had a similar *nodC* sequence to the *Mesorhizobium* isolates [58,60].

Finally, within the IRLC, the pasture legume *Biserrula pelecinus* was introduced into western Australia from the Mediterranean region in 1994 and, as indigenous rhizobial populations in western Australia do not nodulate this legume, the seed was inoculated with *M. ciceri* sv *biserrulae* strain WSM1271 [63,64]. In 2000, *Mesorhizobium* strains, including WSM2073 and WSM2075 with 16S rRNA, *dnaK* and *GS11* phylogenies different from strain WSM1271, were isolated from nodules of *B. pelecinus* grown in Western Australia and shown to nodulate the legume, although the bacteria were largely ineffective with regard to N_2_ fixation [63,64]. Where tested, these strains had identical sequences for the symbiosis insertion regions with WSM1271, indicating that they had obtained their symbiosis genes via horizontal transfer of a symbiosis island from the inoculant within the space of six years. This quick transfer in the field was not a single event, as it was also demonstrated for commercially grown *B. pelecinus* inoculated with WSM1497 and resulted in three different *Mesorhizobium* lineages with identical symbiosis genes to the inoculant [64]. The ability of rhizobial strains that do not fix N_2_ to produce nodules on *B. pelecinus* could result in decreased yield of the crop.

#### 3.2.2. Papilionoideae with Indeterminate Nodules Excluding the IRLC

Phylogenetic incongruence occurs between core and symbiosis genes for *Azorhizobium*, *Bradyrhizobium*, *Burkholderia*, *Ensifer*, *Mesorhizobium*, *Methylobacterium*, *Microvirga*, *Neorhizobium*, *Ochrobactrum*, *Rhizobium*, and *Phyllobacterium* associated with Papilionoideae legumes with indeterminate nodules excluding the IRLC (Table 3). 

Four studies carried out on species within the Crotalarieae found evidence of horizontal transfer of symbiosis genes between different genera of rhizobia. *Rhizobium* isolated from *Aspalathus* sp. grown in the Cape Fynbos biome in South Africa and characterized on 16S rRNA and housekeeping gene sequences had *nifH* and *nodA*,*B*,*C* sequences closely related to those of *Mesorhizobium* [65]. *Methylobacterium nodulans* ORS2060^T^ isolated from *Crotalaria podocarpa* in Senegal, grouped with *Bradyrhizobium* spp. on *nodA* sequences [66]. *Microvirga lotonidis* WSM3557^T^ and *Microvirga zambiensis* WSM3693^T^ isolated from *Listia angolensis* in Zambia had identical *nodA* sequences which clustered with strains of *Bradyrhizobium*, *Burkholderia*, and *Methylobacterium* [67]. *Burkholderia*, isolated from *Rafnia triflora* in the Core Cape subregion of South Africa and characterized on 16S rRNA and housekeeping gene sequences, had a *nifH* sequence closely related to those of *Ensifer* spp. [68]. 

Lemaire and co-workers specifically assessed the degree of horizontal transfer of nodulation genes within rhizobia genera of a range of legumes endemic to the Cape Fynbos biome in South Africa [65]. It was concluded that *Mesorhizobium* strains isolated from *Aspalathus* spp., *Argyrolobium* spp. (Genisteae, Table 3), *Otholobium* spp., and *Psoralea* spp. (Psoraleeae, Table 4), and *Burkholderia* isolated from *Podalyria calyptrata* (Table 3) show high degrees of horizontal transfer of nodulation genes among closely related species. In associated studies, a *Mesorhizobium* isolate from *Psoralea* sp., characterized on 16S rRNA and housekeeping gene sequences, aligned closely to *Ensifer* on *nifH* sequence, and a *Mesorhizobium* isolate from *Psoralea oligophylla* aligned closely to *Burkholderia* on *nodA* sequence (Table 4) [68]. Also, for *Burkholderia* isolated from *P. calyptrata*, different branching patterns were found among numerous isolates for *recA* and *nodA* phylogenies [83]. In a separate study of *Burkholderia* isolates from *Hypocalyptus* spp. (Hypocalypteae) and *Cyclopia* spp., *P. calyptrata* and *Virgilia oroboides* (Podalyrieae) sampled in the Cape Floristic Region of South Africa, phylogenies inferred from *nifH* and *nodA* sequences were incongruent and, generally, phylogenies inferred from *nifH* and *nodA* sequences were incongruent with those from 16S rRNA and *recA* sequences [79]. These findings confirm that horizontal transfer of symbiosis genes is common in South African *Burkholderia*, which is in contrast with findings for South American *Burkholderia* rhizobial symbionts [37,40].

There is also strong evidence that horizontal transfer of symbiosis genes has occurred between different *Bradyrhizobium* spp. associated with *Cytisus* spp. [69,70,71], *Genista versicolor* [72] and *Lupinus* spp. [73,74,75,77,78] in the tribe Genisteae (Table 3). In particular, most *Bradyrhizobium* isolates from native *Lupinus* spp. (and native Genisteae species in general [90]) in Europe form a distinct lineage, ‘Clade II’, on the basis of their *nodA* gene sequences [75,81]. Also, *Bradyrhizobium* isolates from native *Cytisus villosus* in Morocco had diverse 16S rRNA and housekeeping gene sequences, but all showed similar *nifH* and *nodC* sequences which were closely related to those of *Bradyrhizobium japonicum* sv. *genistearum* [71]. These findings indicate that horizontal transfer of symbiosis genes has played a role in the development of specific relationships between Genisteae spp. and *Bradyrhizobium* spp. over wide areas. *Bradyrhizobium* isolated from invasive *Cytisus scoparius* in the United States had housekeeping genes similar to indigenous *Bradyrhizobium*, but their *nodC*, *nifD*, and *nifH* sequences were highly similar or identical to those of a *Bradyrhizobium* strain from Spain [70]. It appears, therefore, that indigenous North American *Bradyrhizobium* had acquired symbiosis genes from *Bradyrhizobium* symbionts of European *C. scoparius* via horizontal gene transfer.

*Microvirga* and *Ochrobactrum*, which are rare as rhizobial symbionts, can nodulate specific *Lupinus* spp. [17,67,76]. The *nifD* and *nifH* sequence for *Microvirga lupini* Lut6^T^ isolated from *Lupinus texensis* aligned closely to *Rhizobium etli* CFN 42^T^, while its *nodA* sequence was placed in a clade that contained strains of *Rhizobium*, *Mesorhizobium*, and *Ensifer* [67]. The *nifH* sequence for *Ochrobactrum lupini* LUP21^T^ isolated from *Lupinus honoratus* in Argentina showed 99.6% similarity to two *Mesorhizobium* strains, while its *nodD* sequence showed 86.4% similarity to *R. etli* CFN 42^T^ [76]. Thus, evidence is strong that *M. lupini* Lut6^T^ and *O. lupini* LUP21^T^ obtained their symbiosis genes from other, more common rhizobial genera.

Incongruence between housekeeping and symbiosis genes indicate that horizontal gene transfer of symbiosis genes has occurred between *Mesorhizobium* strains from *Coronilla varia* (Loteae) grown in Shaanxi province, China [80], *Bradyrhizobium* isolates from *Ornithopus* spp. (Loteae) sampled in Europe and western Australia [75,81], and *Ensifer* strains from *Tephrosia* spp. (Millettieae) in the Indian Thar Desert [82]. New Zealand endemic *Sophora* spp. are nodulated by diverse *Mesorhizobium* spp. with similar symbiosis genes (Figure 1) [85,86,91,92]. Generally, *Mesorhizobium* isolates from the same field site grouped together on housekeeping gene sequences [85,86]. This apparent link between housekeeping gene sequences and field site in association with almost identical symbiosis genes is consistent with the proposal that horizontal transfer of symbiosis genes to *Mesorhizobium* strains adapted to local soil conditions has occurred, but this requires further testing. The relationship between New Zealand endemic *Sophora* species and *Mesorhizobium* with particular symbiosis genes is highly specific and contrasts with findings for *Sophora alopecuroides* and *Sophora. flavescens* sampled in China, which are nodulated by *Ensifer*, *Mesorhizobium*, *Phyllobacterium*, and *Rhizobium* with a wide range of *nodC* and *nifH* gene sequences [87,93]. The data indicate that symbiosis genes of rhizobia associated with *S. alopecuroides* and *S. flavescens* are primarily maintained by vertical transfer, but there is also evidence for occasional horizontal gene transfer of symbiosis genes within and between rhizobia genera associated with these legume species [87,93].

Horizontal transfer of symbiosis genes has occurred across genera in the case of *Agrobacterium* sp. strain IRBG74 originally isolated from the aquatic legume *Sesbania cannabina* and subsequently shown to effectively nodulate *Sesbania sesban* and seven other *Sesbania* species [21]. Housekeeping gene sequences identified strain IRBG74 as a close relative of the plant pathogen *Agrobacterium radiobacter* (=*Agrobacterium tumefaciens*). However, it did not contain *vir* genes but harboured a sym-plasmid containing *nifH* and *nodA* genes with sequences similar to those of *Ensifer* spp. which nodulate *S. cannabina*. In a separate study, *Rhizobium*/*Agrobacterium* and *Ensifer* isolates from *S. cannabina* with diverse housekeeping gene sequences had similar *nifH* and *nodA* sequences, and one *Rhizobium*/ *Agrobacterium* strain showed highly similar *nifH* and *nodA* sequences to strain IRBG74 [84].

Within the Thermopsideae, *Ammopiptanthus nanus* and *Ammopiptanthus mongolicus* sampled across nine sites in three regions of China were nodulated by *Ensifer*, *Neorhizobium*, *Pararhizobium*, and *Rhizobium* [88]. For strains characterized to species level on 16S rRNA and housekeeping gene sequences, *Ensifer arboris* and *Neorhizobium galegeae* strains aligned with *Ensifer meliloti* ATCC9930^T^ on *nifH* and *nodC* gene sequences, *Phyllobacterium giardinii* strains aligned with *R. leguminosarum* sv. *viciae* USDA 2370^T^ on *nifH* and *nodC* gene sequences, and *R.*/*A. radiobacter* strains aligned with *E. fredii* USDA 205^T^ on *nifH* and *nodC* gene sequences [88]. For *Anagyris latifolia* grown in soil samples collected from within natural populations of the legume growing in the Canary Islands, *Mesorhizobium* isolates with diverse 16S–23S rDNA ITS, 16S rRNA and *glnII* gene sequences had identical *nodC* sequences closely related to *Mesorhizobium tianshanense* USDA 3592^T^ [89].

#### 3.2.3. Papilionoideae with Determinate Nodules

Phylogenetic incongruence between core and symbiosis genes has been described for *Bradyrhizobium*, *Ensifer*, *Mesorhizobium*, *Microvirga*, *Pararhizobium*, *Phyllobacterium*, and *Rhizobium* associated with Papilionoideae legumes with determinate nodules (Table 4). 

Considering crop legumes with determinate nodules, incongruence between housekeeping and symbiosis genes indicates that within-genus horizontal transfer of symbiosis genes has occurred between *Bradyrhizobium* spp. associated with *Arachis hypogaea* (peanut) [94,95], *Bradyrhizobium*, and *Ensifer* spp. associated with *G. max* (soybean) [97,98,99,100] and *Vigna unguiculata* (cowpea) [110,112], *Rhizobium* spp. associated with *P. vulgaris* (common bean) [103,105,106] and *Mesorhizobium* associated with *Lotus corniculatus* (bird’s-foot trefoil) [19,20]. The data indicate that, in particular cases, symbiosis genes of rhizobia associated with all these species have transferred to indigenous soil bacteria. The transfer of symbiosis genes from *Mesorhizobium loti* used as inoculum on *L. corniculatus* in New Zealand to indigenous *Mesorhizobium* strains has been studied in detail [19,20]. Here, it was shown that the chromosomal symbiotic element of *M. loti* ICMP 3153 is transferrable between *Mesorhizobium* strains and can be fully functional in the recipient strain. For *G. max* in Brazil, an indigenous *Bradyrhizobium elkanii* strain and an indigenous *Ensifer fredii* strain had similar *nifH*, *nodC*, and *nodY-nodA* gene sequences to those of *B. japonicum* used as inoculant. This is strong evidence that horizontal transfer of symbiosis genes had occurred between *Bradyrhizobium* inoculum and indigenous strains of *Bradyrhizobium* and *Ensifer* [97]. An *Ensifer* isolate from *G. max* in north-eastern Afghanistan had identical *nodD1* and *nifD* sequences to those of *Bradyrhizobium yuanmingense* [101].

For *P. vulgaris*, strains of *R. leguminosarum* sv. *phaseoli*, *R*. *gallicum*, and *Pararhizobium giardinii*, isolated from plants grown in France, and *R*. *etli*, isolated in Mexico and Belize, had highly similar *nodC* sequences, and a strain characterized as *Rhizobium* on its 16S rRNA sequence aligned with *E. meliloti* on its *nodC* sequence [103]. *R. lusitanum* P1–7T isolated from *P. vulgaris* in Portugal had *nifH* and *nodD* sequences similar to *R. tropici* CIAT 899^T^ and *D. neptuniae* LMG 21357^T^ [104]. In a related study, a *Pararhizobium giardinii* strain characterized on 16S rRNA and housekeeping gene sequences aligned with *Ensifer* spp. on *nodC* sequence [107].

Data are presented for three *Vigna* crop species (Table 4). For *Vigna. angularis* (adzuki bean), grown in the sub-tropical region of China, *Bradyrhizobium*, and *E. fredii* were major, and *Rhizobium*, *Mesorhizobium*, and *Ochrobactrum* were minor rhizobial symbionts [108]. Here, 16S rRNA, housekeeping and *nodC* gene phylogenies were congruent, except that one *Rhizobium* strain aligned with *E. fredii* strains on *nodC* sequences. In a related study on *Bradyrhizobium* isolates from *V. radiata* (mungbean) and *V. unguiculata* grown in subtropical China, 16S rRNA, housekeeping and *nodC* gene phylogenies were mainly congruent [116]. Similarly, for *V. radiata* grown in three agro-ecological regions in Nepal, *Bradyrhizobium* 16S–23S RNA IGS, 16S rRNA, *nodA*, *nodD1*, and *nifD* sequences were mainly congruent, but five *Bradyrhizobium* strains aligned with *E. meliloti* on *nodA* sequences [109]. In contrast, overall phylogenies for core and nodulation genes for *Bradyrhizobium*, isolated from *V. unguiculata* at a range of sites in Botswana and Roodeplaat, South Africa, were incongruent [110]. It was concluded that horizontal gene transfer has significantly influenced the evolution of *V. unguiculata* root-nodule bacteria in these African countries [110]. Also, for *M. vignae* BR3299^T^ isolated from *V. unguiculata* in north-east Brazil, the *nifH* gene sequence aligned with *Mesorhizobium* and *Rhizobium* strains, which supports the proposal that *M. vignae* BR3299^T^ obtained its *nifH* gene via horizontal transfer from *Rhizobium* [111]. 

Examples of horizontal transfer of symbiosis genes within and between rhizobial genera were also found for non-crop species with determinate nodules. For *Desmodium* spp. (Desmodieae) growing in Panxi, Sichuan, China, *nodC* sequences for one *Pararhizobium*, one *Rhizobium*/*Agrobacterium*, and two *Rhizobium* isolates characterized on 16S rRNA and housekeeping gene sequences were identical to those for *Ensifer* strains isolated from *L. leucocephala* in China (Panxi) and Brazil [96]. It was concluded that horizontal transfer of *nodC* genes had occurred between the different genera, but that further work was required to determine the direction of gene transfer [96]. Evidence was also found for lateral transfer of symbiosis genes between rhizobial genera associated with *G. soja* (‘wild soybean’). Here, *Ensifer* and *Rhizobium* isolates characterized on 16S rRNA and housekeeping gene sequences formed a single *Ensifer* lineage on *nifH* and *nodA* sequences [102]. 

Across two studies, diverse *Mesorhizobium* isolated from seven *Lotus* species in the Canary Islands and characterized on 16S rRNA, *atpD*, and *recA* gene sequences clustered together close to *M*. *loti* on *nodC* sequences [113,114]. Similarly, diverse *Mesorhizobium* (16S rRNA) isolated from *L. tenuis* grown in three soils of the Salado River Basin Buenos Aires Province, Argentina, clustered together in a large clade on *nifH* and *nodC* sequences, again close to *M*. *loti* [115]. An *Aminobacter* (Phyllobacteriaceae) strain was also reported to nodulate *L. tenuis* and have a *nodC* sequence similar to the *Mesorhizobium* strains. This is the first report of an *Aminobacter* rhizobial strain, however, and requires verification. Similarly, *Geobacillus* (Phylum Firmicutes), *Paenibacillus* (Firmicutes), and *Rhodococcus* (Actinobacteria) were reported as rhizobial symbionts of *L. corniculatus* [117]. It was stated that these bacterial species had similar *nodA* gene sequences to *Mesorhizobium* isolates from the same plants and that they had obtained their *nodA* gene via horizontal transfer from the *Mesorhizobium*. This report also needs to be verified using authentification experiments and whole-genome sequencing.

## 4. Recombination of Symbiotic Islands

Incongruence between rhizobium core and symbiosis genes is strong evidence that horizontal transfer of symbiosis genes has occurred between strains, but it tells little about when the transfer took place. However, there are several cases linked to rhizobia associated with invasive species or use of rhizobial inoculum on crop species where the transfer of symbiosis genes between rhizobial species has been monitored. Here, we briefly consider the recombination of symbiotic islands and focus on two studies in which symbiosis genes were shown to transfer between a *Mesorhizobium* strain used as a crop inoculant and indigenous soil *Mesorhizobium*. The structure and physiology of *R. leguminosarum* symbiotic plasmids is considered in a separate paper in this Special Issue of Genes [118]. 

**Figure 2:**
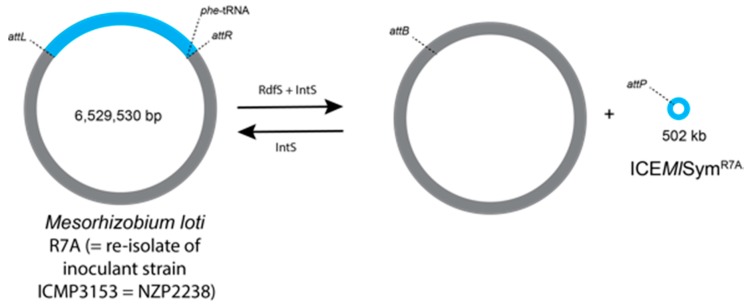
Recombination and circularization of *Mesorhizobium loti* R7A integrative and conjugative elements (ICE*Ml*Sym^R7A^) [20,119,120,121]. The recombination is initiated by IntS (integration factor S) at the attachment sites *attB* and *attP* to integrate the ICE*Ml*Sym^R7A^ in the chromosome and produce the attachment sites *attL* and *attR*. The recombination directionality factor S (RdfS) together with IntS stimulates excision of the ICE and forms the attachment sites *attP* and *attB*. phe-tRNA = phenylalanine transfer RNA. The ICE*Ml*Sym^R7A^ is coloured blue, and the remaining chromosome is coloured grey. The schematic diagram is not drawn to scale.

Firstly, in an important early study, the chromosomal symbiotic element of *M. loti* strain ICMP 3153 used as inoculum on *L. corniculatus* in New Zealand was shown to have transferred to indigenous *Mesorhizobium* strains and was fully functional in the recipient strains [19,20]. Subsequent work on *M. loti* strain R7A, a derivative of strain ICMP 3153, has shown that the symbiosis island is a single 502 kb Integrative Conjugative Element (ICE*Ml*Sym^R7A^) which integrates into a phenylalanine transfer RNA (phe-tRNA) gene [119,120,121] (Figure 2). The recombination reaction is driven by the recombination directionality factor S (RdfS) on the attachment sites *attL*_S_ and *attR*_S_, resulting in the excised ICE*MI*Sym^R7A^. The *attB*_S_ and *attP*_S_ sites facilitate the reintegration of the ICE into the chromosome, driven by integration factor S (IntS). In a separate study, the full genome sequence was reported for *M. ciceri* strain CC1192, which is the sole inoculant used on *C. arietinum* in Australia [122]. The *nod*, *nif*, and *fix* genes appear to be located on a 419 kb symbiosis island integrated within the chromosome of strain CC1192. This symbiosis island shows a similar structure to that of *M. loti* R7A strain.

**Figure 3:**
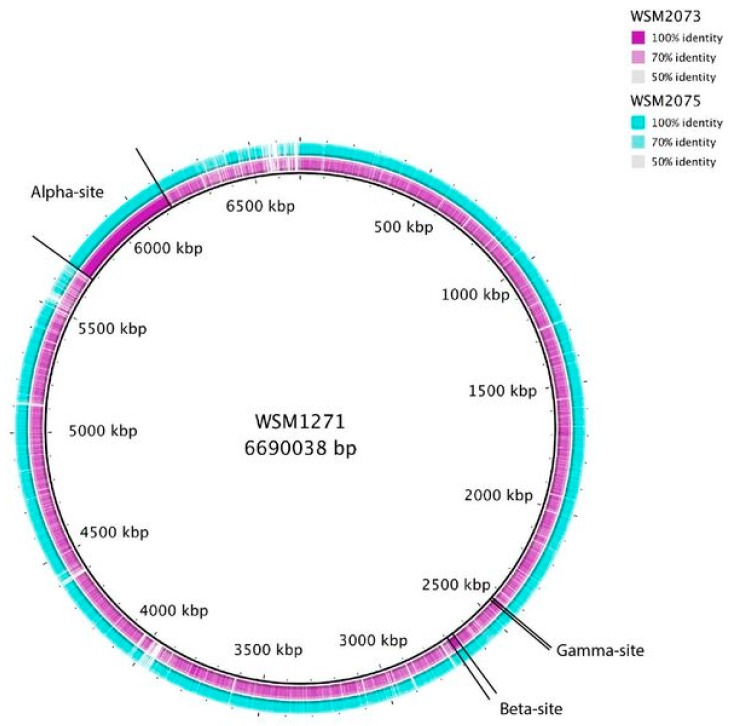
Comparative genome analysis using the circular BLASTN alignment in BRIG (BLAST Ring Image Generator, [124] of WSM2073 and WSM2075 against WSM1271. The three ICE*Mc*Sym^1271^ regions are indicated and summarised from Haskett et al. [123]. It highlights the transfer of the symbiosis island from the *Biserrula pelecinus* inoculant strain WSM1271 to non-symbiotic recipient strains that turn into poorly effective symbionts. ICE*Mc*Sym^1271^ consists of three separate regions (alpha, beta, and gamma) when integrated in the chromosome but excises as one circular plasmid and re-integrates in the recipient chromosome [123].

The second example is the chromosomal symbiotic element of *M. ciceri* sv biserrula strain WSM1271, which was used as inoculum on *B. pelecinus* in western Australia. Again, the symbiotic island was shown to have been transferred from the inoculum to indigenous *Mesorhizobium* strains, but, here, the recipient strains were ineffective or poorly effective on N_2_ fixation of the host plant [63,64]. In this case, the symbiosis ICE exists as three separate chromosomal regions when integrated in their host (alpha, beta, and gamma in Figure 3) [121,123]. These regions occupy three different recombinase attachment sites that do not excise independently but recombine in the host chromosome to form a single contiguous region prior to excision and conjugative transfer. Nine additional tripartite ICEs were identified in diverse mesorhizobia, and transfer was demonstrated for three of them [123]. Single-part ICE and tripartite ICEs appear to be widespread in the *Mesorhizobium* genus [122,123].

## 5. Occurrence and Importance of Horizontal Transfer of Rhizobial Symbiosis Genes

Andrews and Andrews [17] reviewed the literature on legume–rhizobia symbioses in field soils and related genotypically characterized rhizobia (genus level) to the taxonomy of the legumes (species level) from which they were isolated. Symbioses were described for approximately 450 legume species over 255 separate studies, and phylogenetic incongruence between rhizobium core and symbiosis genes was reported in 73 (~30%) of these studies (Table 1, Table 2, Table 3 and Table 4). In the current review, we have listed examples of phylogenetic incongruence between rhizobium core and symbiosis genes for strains of 14 of the currently accepted 15 genera of rhizobia. The exception is *Allorhizobium*, for which no sequences for symbiosis genes are available for comparison with those in the databases. Thus, horizontal transfer of symbiosis genes between rhizobial strains is of common occurrence and is not restricted to specific rhizobial genera.

Phylogenetic comparisons of gene sequences indicated that horizontal transfer of symbiosis genes was more common within than between genera. In several cases such as *Bradyrhizobium* associated with native Genisteae species in Europe [90] and *Mesorhizobium* associated with *Sophora* spp. throughout New Zealand [85,86], within-genus horizontal transfer of *nod* genes has occurred across many bacterial species and is associated with legume divergence over a wide range of habitats and over long time periods. In 27 studies (~35% of those listed here), gene sequences indicated that horizontal transfer of symbiosis genes had occurred between different rhizobia genera. In most cases, this involved gene transfer between the common alphaproteobacterial genera *Bradyrhizobium*, *Ensifer*, *Mesorhizobium*, and *Rhizobium*. However, evidence is strong that these genera provided the symbiosis genes for the less common rhizobia genera *Devosia* [33], *Methylobacterium* [66], *Microvirga* [67,111], and *Ochrobactrum* [76], which thrive under particular environmental conditions. It seems certain that within- and between-genera horizontal transfer of symbiosis genes to indigenous soil bacteria has aided the diversification and establishment of legumes in different habitats.

The data indicate that horizontal transfers of symbiosis genes between alpha- and beta-proteobacteria and between the beta-proteobacteria *Burkholderia* and *Cupriavidus* are of rare occurrence. Only two cases (in one study) were found for horizontal transfer of symbiosis genes between alpha- and beta-proteobacteria. Specifically, in the Core Cape subregion of South Africa, *Burkholderia* isolated from *R. triflora* had a *nifH* sequence closely related to those of *Ensifer* spp., and a *Mesorhizobium* isolate from *P. oligophylla* aligned closely to *Burkholderia* on *nodA* sequence [68]. There are no confirmed reports of horizontal transfer of symbiosis genes between *Burkholderia* and *Cupriavidus*, which may at least in part be related to the genera not overlapping extensively in their biogeographic ranges [17,29,38]. For *Burkholderia*, findings indicate that within-genus horizontal transfer of symbiosis genes is rare for South American species but common in South African species [37,40,65,79]. The reasons for this regional difference are not clear, but the patterns across different rhizobial genera indicate that ‘lateral transfer of symbiosis traits is an important evolutionary force among rhizobia of the Cape Fynbos biome’ [65]. The potential rapidity of these evolutionary-scale changes in bacterial genomes was illustrated by the example from western Australia, where populations of indigenous *Mesorhizobium*, originally unable to nodulate the pasture legume *B. pelecinus*, were isolated from nodules and displayed symbiosis genes obtained from the commercial inoculant within six years [63,64].

In relation to the legume host, horizontal transfer of symbiosis genes to rhizobia and non-rhizobial bacteria that are adapted to local soil conditions is likely to increase the likelihood of establishment of a successful legume–rhizobia symbiosis in these soils as long the recipient bacteria can induce functional N_2_-fixing nodules on the legume. The ecological success of the transfer is enhanced by the strong selection the plant exerts towards efficient infection and nodulation and the action of error-prone DNA polymerases that accelerate adaptation to symbiosis after gene transfer [22]. 

By considering all of the examples described here, we can conclude that horizontal transfer of symbiosis genes between rhizobial strains is of common occurrence, is widespread geographically, is not restricted to specific rhizobial genera, and occurs within and between rhizobial genera. The transfer of symbiosis genes to bacteria adapted to local soil conditions can allow these bacteria to become rhizobial symbionts of previously incompatible legumes growing in these soils. This, in turn, will have consequences for the growth, life history, and biogeography of the legume species involved, which provides a critical ecological link connecting the horizontal transfer of symbiosis genes between rhizobial bacteria in the soil to the above-ground floral biodiversity and vegetation community structure.

## Figures and Tables

**Table 1:** Reported cases of phylogenetic incongruence between core and symbiosis genes for rhizobia associated with legumes in the sub-family Caesalpinioideae. All species have indeterminate nodules.

Caesalpinioideae Mimosoid Clade	Rhizobia
*Acaciella angustissima*	*Ensifer chiapanecum* ITTG S70^T^ and *Ensifer mexicanum* ITTG R7^T^ had different *gyrA*, *nolR*, *recA*. *rpoB*, and *rrs* gene sequences but similar *nifH* and *nodA* sequences [27]
*Leucaena leucocephala*	*Ensifer* isolates formed three clades in both 16S rRNA and *recA* phylogenetic trees but only one clade in both *nifH* and *nodC* trees [28]
*Mimosa cruenta*, *Mimosa magentea*, *Mimosa ramulosa*, *Mimosa reptans*, *Mimosa schleidenii*	*Cupriavidus* isolates separated into two groups on 16S rRNA, *recA* and *gyrB* sequences but grouped together on *nifH* and *nodA* sequences [29]
*Mimosa diplotricha*	*Burkholderia caribensis* TS182 characterized on 16S rRNA sequence grouped with *Cupriavidus* strains on *nodA* sequence [30]
*M. diplotricha*, *Mimosa pudica*	*Burkholderia* strains with diverse 16S rRNA gene sequences grouped together along with *B*. *phymatum* STM815^T^ on *nodA* sequence [31]
*M. pudica*	*Rhizobium altiplani* BR 10423^T^ had *nifH* and *nodC* sequences closely related (identical for *nodC*) to those of *Rhizobium mesoamericanum* CCGE 501^T^ [32]
*Neptunia natans*	*Devosia* isolates characterized on 16S rRNA sequences had *nifH* and *nodD* sequences closely related to those of *Rhizobium tropici* CIAT899^T^ [33]
*Vachellia jacquemontii*	*Ensifer* showed incongruence across all three of concatenated *rrs*-*glnII*-*atpD*-*recA*-*dnaK*, *nifH*, and *nodA* gene sequences [34]
*Vachellia macracantha*	*Ensifer* sequences for *nifH* and *nodC* were incongruent with those for 16S rRNA [35]
*Vachellia seyal*, *Vachellia tortilis*	*Ensifer* isolates separated into seven groups on the basis of 16S rRNA, *recA*, *gyrB*, *rpoB*, *atpD*, *gap* and *pnp* gene sequences but were closely related with respect to their *nifH* and *nodC* gene sequences [36].

**Table 2:** Reported cases of phylogenetic incongruence between core and symbiosis genes for rhizobia associated with legumes in the inverted repeat-lacking clade (IRLC) of the legume sub-family Papilionoideae. All species have indeterminate nodules.

Papilionoideae Tribes and Genera	Rhizobia
**Cicereae**	
*Cicer arietinum*	*Mesorhizobium ciceri*, *Mesorhizobium mediterraneum*, *Mesorhizobium muluense* and *Mesorhizobium* spp. with diverse 16S rDNA, *recA*, *atpD*, *glnII* and *gyrB* sequences had similar *nifH*, *nodA* and *nodC* sequences [42,43,44,45,46,47]
*Cicer canariense*	*Mesorhizobium* with diverse 16S rRNA, *recA* and *glnII* sequences had similar *nodC* gene sequences [48]
**Fabeae**	
*Lathyrus* spp., *Lens culinaris*, *Pisum sativum* and *Vicia* spp.	*Rhizobium fabeae*, *Rhizobium pisi*, *Rhizobium laguerreae*, *Rhizobium anhuiense*, *Rhizobium bangladeshense*, *Rhizobium binae*, *Rhizobium lentis* and *Rhizobium* spp. with diverse 16S rRNA and *recA*, *atpD* and *glnII* sequences had similar *nifH*, *nodA* and *nodC* sequences [49,50,51,52,53,54]
*Lathyrus odoratus*, *Vicia hirsuta*	*Rhizobium multihospitium* isolates had *nifH* and *nodD* sequences 100% similar to those of *Rhizobium lusitanum* P1–7^T^ and *Devosia neptuniae* J1^T^ [55]
**Galegeae**	
*Astragalus aksuensis*, *Astragalus* sp., *Oxytropis glabra*, *Oxytropis meinshausenii*	*R. multihospitium* isolates had *nifH* and *nodD* sequences 100% similar to those of *R. lusitanum* P1–7^T^ and *D. neptuniae* J1^T^ [55]
*Astragalus glycyphyllos*	*Mesorhizobium* isolates showing 16S rRNA sequences similar to *M*. *ciceri*, *Mesorhizobium amorphae* or *Mesorhizobium septentrionale* formed one clearly separated, closely related cluster for *nodA*, *nodC*, *nodH* and *nifH* sequences [56]
*Glycyrrhiza uralensis*	*Mesorhizobium* concatenated *rrs-recA-rpoB*, *nifH*, *nodA* and *nodC* sequences were not congruent [57]
*Sphaerophysa salsula*	*Mesorhizobium* with diverse 16S rRNA sequences showed similar *nifH* sequences [58]*Mesorhizobium* and *Rhizobium* identified on 16S rRNA sequences showed similar *nifH* sequences [58]
**Hedysareae**	
*Alhagi* sparsifolia	*Mesorhizobium* isolates separated into three groups on the basis of their *rrs*, *dnaK* and *dnaJ* sequences but their *nodA* and *nodC* sequences were closely related [59]
*Alhagi* sp., *Caragana jubata*, *Halimodendron halodendron*,	*R. multihospitium* isolates had *nifH* and *nodD* sequences 100% similar to those of *R. lusitanum* P1-7^T^ and *D. neptuniae* J1^T^ [55]
*Caragana bicolor*, *Caragana erinacea*, *Caragana franchetiana*, *Caragana intermedia*	*Mesorhizobium* isolates with diverse 16S–23S IGS 16S rRNA sequences and one *Bradyrhizobium* isolate (16S rRNA) from *C*. *intermedia* had similar *nodC* sequences [60]
*Trifolium*	
*Trifolium repens*	*R. pisi* sv. *trifolii* K3.22 characterised on the basis of 16S rRNA, *atpD*, *dnaK*, *glnA*, *gyrB*, *recA* and *rpoB* sequences had *nodA*, *nodB*, *nodC* and *nodD* sequences with high similarity to those of *Rhizobium leguminosarum* sv. *trifolii* [61].*Rhizobium* spp. with diverse 16S rRNA and concatenated *atpD-recA-glnII* sequences had similar *nifH* and *nodC* sequences [62]

**Table 3:** Legume–rhizobia symbioses of species in the sub-family Papilionoideae with indeterminate nodules excluding the IRLC.

Papilionoideae Tribes (Genera)	Rhizobia
**Crotalarieae**	
*Aspalathus* sp.	*Rhizobium* isolate characterized on 16S rRNA and concatenated *recA*-*atpD*-*gyrB*-*glnA* sequences had *nifH* and concatenated *nodA-B-C* sequences closely related to those of *Mesorhizobium* [65]
*Aspalathus astroites*, *Aspalathus aurantiaca*, *Aspalathus bracteata*, *Aspalathus ciliaris*, *Aspalathus cordata*, *Aspalathus ericifolia*, *Aspalathus spicata*	*Mesorhizobium* phylogenetic relationships between concatenated *recA*-*atpD*-*gyrB*-*glnA* and *nodA-B-C* sequences were incongruent [65]
*Crotalaria podocarpa*	*Methylobacterium nodulans* ORS2060^T^ *nodA* sequence groups with *nodA* sequences for *Bradyrhizobium* spp. [66]
*Listia angolensis*	*Microvirga lotonidis* WSM3557^T^ and *Microvirga zambiensis* WSM3693^T^ *nodA* sequences were identical and clustered with *Bradyrhizobium*, *Burkholderia* and *Methylobacterium nodA* sequences [67]
*Rafnia triflora*	*Burkholderia* isolate characterized on concatenated 16S rRNA-*recA-atpD* sequences had a *nifH* sequence closely related to those of *Ensifer* spp. [68]
**Genisteae**	
*Argyrolobium lunare*, *Argyrolobium velutinum*	*Mesorhizobium* phylogenetic relationships between concatenated *recA*-*atpD*-*gyrB*-*glnA* and *nodA-nodB-nodC* sequences were incongruent [65]
*Cytisus proliferus*	*Bradyrhizobium* with diverse 16S–23S rRNA, *atpD*, *glnII* and *recA* sequences showed similar *nifH* and *nodC* sequences [69]
*Cytisus scoparius*	*Bradyrhizobium* 16S rRNA, 23S rRNA, *dnaK*, *gyrB*, *rplC*, *rpoB*, *nifD*, *nifH* and *nodC* sequences indicated a highly heterogeneous ancestry [70]
*Cytisus villosus*	*Bradyrhizobium* with diverse 16S rRNA and concatenated *glnII-recA* sequences showed similar *nifH* and *nodC* sequences [71]
*Genista versicolor*	*Bradyrhizobium* with diverse 16S–23S ITS and *atpD* sequences showed similar *nifH* and *nodC* sequences for almost all strains [72]
*Lupinus albus*	*Bradyrhizobium* with diverse 16S–23S ITS and *rrs* and *atpD* sequences clustered together on *nodC* sequences [73]
*L. albus*, *Lupinus angustifolius*, *Lupinus luteus*, *Lupinus* sp.	*Bradyrhizobium* with diverse 16S–23S ITS and 16S rRNA sequences clustered together on *nodC* sequences [74]
*L. albus*, *L*. *angustifolius*, *L*. *luteus*	*Bradyrhizobium* with diverse concatenated *atpD*-*glnII*-*recA* sequences clustered together on *nodA* sequences [75]
*Lupinus honoratus*	*Ochrobactrum lupini* LUP21^T^ *nifH* sequence showed 99.6% similarity to *M. ciceri* strains; its *nodD* sequence showed 86.4% similarity to *Rhizobium etli* CFN42^T^ [76]
*Lupinus mariae-josephae*	*Bradyrhizobium* with diverse concatenated *atpD*-*glnII*-*recA* sequences separated into two distinct clusters on *nodA* and *nodC* sequences [77]
*Lupinus micranthus*	*Bradyrhizobium* with diverse concatenated 16S rRNA and concatenated *atpD-gln11-recA* sequences showed similar *nodC* gene sequences [78]
*Lupinus texensis*	*Microvirga lupini* Lut6^T^ concatenated *nifD-nifH* sequence aligned close to *R. etli* CFN42^T^; its *nodA* sequence was placed in a clade that contained strains of *Rhizobium*, *Mesorhizobium* and *Ensifer* [67]
**Hypocalypteae**	
*Hypocalyptus sophoroides*, *Hypocalyptus oxalidifolius*, *Hypocalyptus colutoides*	*Burkholderia* phylogenies inferred from *nifH* and *nodA* sequences were incongruent; *Burkholderia* phylogenies inferred from *nifH* and *nodA* sequences were incongruent with those from 16S rRNA and *recA* sequences [79]
**Loteae**	
*Coronilla varia*	*Mesorhizobium* phylogenies for 16S rRNA, *nifH* and *nodC* sequences were incongruent [80]
*Ornithopus compressus*, *Ornithopus sativus*	*Bradyrhizobium* with diverse 16S–23S rRNA ITS and dnaK, *atpD*, glnII and *recA* sequences clustered together on *nodA*, *nodZ* and *nolL* sequences [75,81]
**Millettieae**	
*Tephrosia falciformis*, *Tephrosia leptostachya*, *Tephrosia purpurea*, *Tephrosia villosa*, *Tephrosia wallichii*	*Ensifer* 16S rRNA and concatenated *recA-atpD-glnII-dnaK* sequences grouped with *Ensifer saheli* LMG 7837^T^ and *Ensifer kotiensis* LMG 19225^T^ but *nifH*, *nodA* and *nodC* sequences clustered with *Ensifer fredii* USDA 205^T^ [82]
**Podalyrieae**	
*Cyclopia buxifolia*, *Cyclopia genistoides*, *Cyclopia glabra*, *Cyclopia intemedia*, *Cyclopia longifolia*, *Cyclopia maculata*, *Cyclopia meyeriana*, *Cyclopia pubescens*, *Cyclopia sessiflora*, *Cyclopia subternata*	*Burkholderia* phylogenies inferred from *nifH* and *nodA* sequences were incongruent; phylogenies inferred from *nifH* and *nodA* sequences were incongruent with those from 16S rRNA and *recA* sequences [79]
*Podalyria calyptrata*	*Burkholderia* phylogenetic relationships between concatenated *recA*-*atpD*-*gyrB-glnA* and *nodA-B-C* sequences were largely incongruent [65]*Burkholderia* phylogenies inferred from *nifH* and *nodA* sequences were incongruent; phylogenies inferred from *nifH* and *nodA* sequences were incongruent with those from 16S rRNA and *recA* sequences [79]*Burkholderia* phylogenetic relationships between *recA* and *nodA* sequences were largely congruent but different branching patterns were observed among numerous isolates [83]
*Virgilia oroboides*	*Burkholderia* phylogenies inferred from *nifH* and *nodA* sequences were incongruent; phylogenies inferred from *nifH* and *nodA* sequences were incongruent with those from 16S rRNA and *recA* sequences [79]
**Robineae**	
*R. pseudoacacia*	*R. multihospitium* isolates had *nifH* and *nodD* sequences 100% similar to those of *R. lusitanum* P1–7^T^ and *D. neptuniae* J1^T^ [55]
**Sesbanieae**	
*Sesbania cannabina*	*Rhizobium* strain IRBG74 characterised on concatenated 16S rRNA–*rpoB-fusA* sequence harboured a sym-plasmid containing *nifH* and *nodA* genes similar to those of *Ensifer* strains that nodulate this legume [21]*Rhizobium*/*Agrobacterium* and *Ensifer* characterized on concatenated *recA*-*atpD*-*glnII* sequences had similar *Ensifer nifH* and *nodA* sequences [84]
*Sesbania sesban*	*Ensifer* isolates separated into three groups on the basis of concatenated 16S rRNA-*recA*-*gyrB*-*rpoB*-*atpD*-*gap*-*pnp* sequences but were closely related with respect to their *nifH* and *nodC* sequences [36]
**Sophoreae**	
*Sophora alopecuroides*	*R. multihospitium* isolates had *nifH* and *nodD* sequences 100% similar to those of *R. lusitanum* P1–7^T^ and *D. neptuniae* J1^T^ [55]
*Sophora chathamica*, *Sophora fulvida*, *Sophora godleyi*, *Sophora longicarinata*, *Sophora microphylla*, *Sophora prostrata*, *Sophora tetraptera*	*Mesorhizobium* with diverse concatenated *recA-glnII-rpoB* sequences had similar *nifH*, *nodA* and *nodC* sequences [85,86]
*Sophora flavescens*	*Rhizobium mongolense* isolate characterized on concatenated *atpD*-*glnII*-*recA* sequences had *nodC* sequence similar to isolates characterized as *M. septentrionale* [87]. *E. fredii* isolate characterized on concatenated *atpD*-*glnII*-*recA* sequences had *nodC* sequence identical to *Mesorhizobium temperatum*^T^ [87]. *Phyllobacterium sophorae*^T^ isolate characterized on concatenated *atpD*-*glnII*-*recA* sequences had *nodC* sequence closely related to *M. septentrionale*^T^ [87]. *Mesorhizobium* and *Rhizobium* phylogenetic relationships between concatenated *atpD-glnII-recA* and *nodC* sequences were incongruent [87]
**Thermopsideae**	
*Ammopiptanthus nanus*, *Ammopiptanthus mongolicus*	*Ensifer arboris* and *Neorhizobium galegeae* characterized on 16S rRNA and concatenated *recA-atpD-rpoB-thrC* sequences aligned with *Ensifer meliloti* ATCC9930^T^ on *nifH* and *nodC* sequences [88]. *Phyllobacterium giardinii* characterized on 16S rRNA and concatenated *recA-atpD-rpoB-thrC* sequences aligned with *R. leguminosarum* sv. *viciae* USDA 2370^T^ on *nifH* and *nodC* sequences [88]. *Rhizobium*/*Agrobacterium radiobacter* characterized on 16S rRNA and concatenated *recA-atpD-rpoB-thrC* sequences aligned with *E. fredii* USDA205^T^ on *nifH* and *nodC* sequences [88]
*Anagyris latifolia*	*Mesorhizobium* isolates with diverse 16S–23S rDNA ITS, 16S rRNA and *glnII* sequences had identical *nodC* sequences closely related to *Mesorhizobium tianshanense* USDA 3592^T^ [89]

**Table 4:** Legume–rhizobia symbioses of species in the sub-family Papilionoideae with determinate nodules.

Papilionoideae Tribes and Genera	Rhizobia
**Dalbergieae**	
*Arachis hypogaea*	*Bradyrhizobium guangdongense* CCBAU 51649^T^, *Bradyrhizobium guangxiense* CCBAU 53363^T^, *Bradyrhizobium* sp. P1237 and *Bradyrhizobium* sp. CH81 had identical *nodA* sequences [94]; *Bradyrhizobium* with diverse 16S–23S rRNA ITS and concatenated *atpD*-*recA* sequences showed similar *nodA* sequences [95]
**Desmodieae**	
*Desmodium oldhami*	*Rhizobium* characterized on 16S rRNA and concatenated *recA-atpD-glnII* sequences aligned with *Ensifer* sp. on *nodC* sequences [96]
*Desmodium sequax*	*Rhizobium* and *Pararhizobium* characterized on 16S rRNA and concatenated *recA-atpD-glnII* sequences aligned with *Ensifer* sp. on *nodC* sequences [96]
**Phaseoleae**	
*Glycine max*	*Bradyrhizobium* strains with clearly separated 16S rRNA sequences showed identical or similar *nifH*, *nodC* and *nodY-nodA* sequences [97]; *Ensifer* strain characterized on 16S rRNA sequence showed similar *nifH*, *nodC* and *nodY-nodA* sequences to *B. japonicum*^T^ [97]; *Bradyrhizobium* with diverse 16S rRNA and concatenated *recA-glnII-atpD* sequences showed identical *nifH* and *nodC* sequences [98]; *Ensifer* with diverse 16S rRNA and concatenated *recA-glnII-atpD* sequences showed identical *nifH* and *nodC* sequences [98]; *Ensifer sojae* CCBAU 05684^T^ and *E. fredii* USDA 205^T^ showed identical *nodC* sequences [99]; *Bradyrhizobium daqingense* CCBAU 15774^T^, *Bradyrhizobium liaonginenese* USDA 3622^T^ and *B*. *japonicum* USDA 6^T^ showed identical *nifH* and *nodC* sequences [100]; *Ensifer* isolate classified on 16S rRNA sequence showed 99% similarity to *Bradyrhizobium yuanmingense* in *nodD1* and *nifD* sequences [101]
*Glycine soja*	*Ensifer* and *Rhizobium* with diverse 16S rRNA and concatenated *recA-atpD-glnII* sequences formed a single *Ensifer* lineage on *nifH* and *nodA* sequences [102]
*Phaseolus vulgaris*	*R. etli*, *Rhizobium gallicum*, *R*. *leguminosarum* sv. *phaseoli* and *Pararhizobium giardinii* characterized on 16S rRNA sequences had similar *nodC* sequences and a strain characterized as *Rhizobium* aligned with *E. meliloti* on *nodC* sequence [103]; *R. lusitanum* P1–7^T^ had *nifH* and *nodC* sequences similar to *D. neptuniae* LMG 21357^T^ and *R.* tropici CIAT 899^T^ [104]; *R. etli* and *R*. *leguminosarum* characterized on 16S rRNA sequences showed similar *nifH* and *nodC* sequences to *R*. *etli* CFN 42^T^ [105]; *Rhizobium* with diverse 16S rRNA and concatenated *atpD-glnII-recA* sequences clustered together on *nifH* and *nodC* sequences [106]; *Pararhizobium giardinii* characterized on 16S rRNA and concatenated *recA-glnII-atpD* sequences aligned with *Ensifer* on *nodC* sequence [107]
*Vigna angularis*	*Rhizobium* characterized on 16S rRNA and concatenated *atpD-recA* sequences had a *nodC* sequence similar to *Ensifer* strains [108]
*Vigna radiata*	*Bradyrhizobium* characterized on sequences of the 16S rRNA, *nodD1* and *nifD* genes and the ITS region aligned with *Ensifer* on *nodA* sequences [109]
*Vigna unguiculata*	*Bradyrhizobium* with diverse concatenated *rrs-recA-glnII* sequences showed similar *nodA* sequences [110]; *Microvirga vignae* BR3299^T^ aligned with *Mesorhizobium* and *Rhizobium* on *nifH* sequence and *Microvirga lotononidis*, *M. zambiensis*, *Bradyrhizobium*, *Burkholderia* and *Methylobacterium* on *nodA* sequences [111]; *E. fredii* characterized on sequences of 16S rRNA, concatenated *recA-glnII-gyrB-truA-thrA-SMc00019* and IGS were substantially diverged from *E*. *fredii* on *nifH*, *nodC* and *rhcRST-1* sequences [112]
**Psoraleae**	
*Otholobium bracteolatum*, *Otholobium hirtum*, *Otholobium virgatum*, *Otholobium zeyheri*	*Mesorhizobium* phylogenetic relationships between concatenated *recA*-*atpD*-*gyrB*-*glnA* and *nodA-B-C* sequences were incongruent [65]
*Psoralea asarina*, *Psoralea congesta*, *Psoralea laxa*, *Psoralea rigidula*	*Mesorhizobium* phylogenetic relationships between concatenated *recA*-*atpD*-*gyrB*-*glnA* and *nodA-B-C* sequences were incongruent [65]
*P. oligophylla*	*Mesorhizobium* isolate characterized on concatenated 16S rRNA-*recA*-*atpD* sequence aligned closely to *Burkholderia* on *nodA* sequence [68]
*Psoralea* sp.	*Mesorhizobium* isolate characterized on concatenated 16S rRNA-*recA*-*atpD* sequence aligned closely to *Ensifer* on *nifH* sequence [68]
**Loteae**	
*Lotus bertheloti*, *Lotus callis-viridis*, *Lotus corniculatus*, *Lotus campylocladus*, *Lotus pyranthus*, *Lotus sessifolius*, *L*. *tenuis*	*Mesorhizobium* with diverse 16S rRNA, *atpD* and *recA* sequences clustered together on *nodC* gene sequences [113,114]
*L. corniculatus*	Transfer of symbiotic island between *Mesorhizobium loti* inoculum and indigenous *Mesorhizobium* strains [19,20]
*L. frondosus*, *L*. *tenuis*	*R. multihospitium* isolates had *nifH* and *nodD* sequences 100% similar to those of *R. lusitanum* P1–7T and *D. neptuniae* J1^T^ [55]
*L. tenuis*	*Mesorhizobium* with diverse 16S rRNA sequences clustered together on *nifH* and *nodC* sequences [115]
